# Physiotherapy and Rehabilitation in a Child with Joubert Syndrome

**DOI:** 10.1155/2017/8076494

**Published:** 2017-08-23

**Authors:** Özge İpek, Özge Akyolcu, Banu Bayar

**Affiliations:** ^1^Faculty of Health Sciences, Department of Physiotherapy and Rehabilitation, Muğla Sıtkı Koçman University, Muğla, Turkey; ^2^Department of Physiotherapy and Rehabilitation, Private Yedigün Medical Center, İzmir, Turkey

## Abstract

**Objective:**

Joubert syndrome (JS) is a rare autosomal recessive genetic disorder characterized by brain malformation, hypotonia, breathing abnormalities, ataxia, oculomotor apraxia, and developmental delay. The purpose of this study was to report the efficiency of the physiotherapy and rehabilitation program in a child with JS.

**Materials and Methods:**

Our case is a 19-month-old female child with mild clinical signs of JS. The pretreatment and posttreatment motor functioning level of the case was evaluated through the Gross Motor Function Measure (GMFM), whereas the independence level was evaluated through the Pediatric Functional Independence Measure (WeeFIM). The case was included in the rehabilitation program by the physiotherapist for one hour for five days a week throughout the period of 13 months in accordance with the neurodevelopmental treatment principles.

**Results:**

The case was able to turn around from the supine position to the reverse direction by oneself, and she was able to rise on her forearms facedown and was able to sit, crawl, and walk independently. The GMFM score was 210, whereas WeeFIM score was 65.

**Discussion:**

In the direction of those findings, in Joubert Syndrome, physiotherapy and rehabilitation can be effective in coping with the symptoms causing developmental delay.

## 1. Introduction

Joubert Syndrome (JS) is rare genetic heterogeneously inherited neurodevelopmental disorder, which was identified in 4 siblings for the first time in 1969 by Joubert et al. [1, 2]. When JS is caused by mutations in the OFD1 gene on the X chromosome, it is inherited in an X-linked recessive manner [3]. Among its clinical findings are ataxia, abnormal eye movements, hyperpnea episodes, hypotonia, respiratory anomalies, mental retardation, and growth retardation [3–6]. A prevalence of less than 1 in 100,000 and almost 200 cases around the world have been reported [7]. The “molar tooth finding” stated to be the distinctive feature of this syndrome today emerges in the form of midbrain-hindbrain anomalies, and this, in the axial MRI images, involves elongation, thinning, and deepening in the interpedicular fossa in the pontomesencephalic junction due to the dysgenesis of the isthmus region of the cerebral trunk [8–11]. The recent advent of next-generation sequencing strategies has enabled impressive progress in our knowledge of JS, with several genes being identified and characterized at the pathophysiological level. Doherty et al. [3] reported that mutations in 20 genes can cause JS and related disorders in 2013. These genes can be listed as follows: AHI1, ARL13B, C5ORF42, CC2D2A, CEP41, CEP290, INPP5E, KIF7, MKS1, NPHP1, OFD1, RPGRIP1L, TCTN1, TCTN2, TCTN3, TMEM67, TMEM138, TMEM216, and TMEM23 [3]. The gene products function in primary cilia which are the pathogenic basis of this clinically and genetically heterogeneous disorder relates to the dysfunction of a subcellular organelle making these disorders part of an expanding group of disorders called ciliopathies [12, 13]. In embryonic development, increasing evidence suggests a key role for primary cilia in regulating main pathways, such as Shh, Wnt, and planar cell polarity, which are implicated in left–right axis formation, limb development, and neurogenesis [14]. The brain malformations might result from defects in midline fusion of the developing vermis [15] or defects in sonic hedgehog-mediated granule cell proliferation [16, 17] because loss of function in the genes associated with JS disrupts protein localisation to the primary cilium [18] where several neurotransmitter receptors are normally localised [19, 20].

Ferland et al. [21] also looked at the expression of AHI1 in mice to try to better understand its role: “since individuals with JS lack a cerebellar vermis and AHI1 expression occurs very early in mouse cerebellar development, then AHI1 may have an important role in the formation or patterning of the cerebellar vermis.” Although the clinical course of JS, which is suspected of in the first few months of life, is quite unsteady, most children reach adulthood during this process [14, 22]. Children with JS have characteristic facial features including a little wider-than-normal occipital frontal contour, prominent and dominant forehead, more circular eye brows, lowered and thick ears, anteverted nostrils, a large nasal base, and protruded and rhythmic tongue movements [23]. In 2013 Romani et al. [14] reported that, at the neuropathological level, JS results from the hypoplasia and dysplasia of the cerebellar vermis and of pontine and medullary structures, and the absence of decussation of the superior cerebellar peduncles and the pyramidal tracts [24–26]. This failure of selected tracts to cross the brainstem midline has been further confirmed by diffusion tensor imaging studies [27, 28] and implies an underlying defect of axon guidance that is unique to JS among all ciliopathies and still remains unexplained [29]. The treatment of JS is carried out in a rather symptomatic and supportive manner [30, 31]. Special approaches are being practiced for the respiratory and dietary problems that occur in relation to respiratory abnormalities and hypotonia during the early stage [32–35]. Rehabilitation strategies are also shaped in terms of cognitive and behavioural difficulties as well as visual weakness [33].

When reviewing literature, no results of a physiotherapy and rehabilitation program supporting the motor development of the children with JS were available. In this study, it was aimed that the physiotherapy and rehabilitation process of a 13-month case with JS be shared.

The aim of this study presents the results of the 13-month physiotherapy and rehabilitation process in case with JS.

## 2. Case Report

The 19-month-old female case was born at term, 49 cm in height and 2765 gr weight, by normal delivery. That was the first gestation and first delivery of the mother. The gestational age was 28. The mother in question had no serious disease throughout her pregnancy. There is no kinship between the mother and the father. There is nobody else within the family who has this anomaly. The case was diagnosed with JS when she was 4,5 months old. Her mother applied to an ophthalmologist due to the fact that her forty-day baby failed to follow the objects around. On account of the fact that the ophthalmologist told the mother that her baby was yet too little and she would be able to make an eye contact in a few months, the mother waited until the baby was 4 months old. However, seeing that there was no change at all in the baby's condition, she had to apply to a neurologist. In the cranial CT examination of the baby, the cerebral trunk seemed to be in its usual shape, whereas there were findings of deformation in the 4th ventricle, and there was the sight of a molar tooth in the mesencephalon and superior cerebellar peduncle. The superior cerebellar peduncle was found to be thicker than expected. A cleft in the cerebellar vermis drew attention. During the ophthalmologic examination, no nystagmus was found. The mother defined her baby as a “floppy one,” stating that she could not even establish an eye contact with her, that her tongue was always protruded, and that she had no problem with her respiration or her ability to understand. The case started to speak only after reaching the age of 1, and her first sensible word was “mom.” When the case was 12 months old, she was made to start a private educational institution upon the doctor's advice since her motor development had fallen behind that of her peers. As the mother could see the improvement in her child as time went by, she decided to continue physiotherapy and rehabilitation sessions. When the case was 19 months old, she was included in the physiotherapy and rehabilitation program in a private medical center. The case had motor movements such as head control, assisted sitting, and turning right and left before the physiotherapy and rehabilitation program started on. However, there were no such movements as crawling, turning from the supine position back to the sitting position, and staying on forearms in a facedown position. In order to determine the motor functioning level as well as the skill level of the case, the Gross Motor Function Measurement Test (GMFM) was used. This test consists of a total of 88 items along with 5 functional dimensions, which involve lying and rolling (17 items); sitting (20 items); crawling and kneeling (14 items); standing (13 items); and walking, running, and jumping (24 items) that are followed by one another consecutively in neurological development order. The test in question was standardized in the way that it would indicate the developments in time. The scoring of each item is performed according to the likert scale. If the activity cannot be started on, then “0” point is given, whereas if it is started independently, then “1” point is given; on the other hand, if the activity is partially completed, then “2” points are given, and if it is completed independently, then “3” points are given. The maximum scores to be obtained from the sections are as follows: lying and rolling: 51; sitting: 60; crawling and kneeling: 42; standing: 39; and walking, running and jumping: 72. The total score obtained in each section is divided by the overall score of that section and is then multiplied by 100, as a result of which the total score for that section (%) is calculated. The calculation for the total score is obtained by dividing the sum of these 5 sections by 5 [36, 37].

Our case got 20 points from the lying and rolling section of the test (Table 1). While in this section, she got the overall score from the items involving the full joint range of motion and flexion of right and left hips and knee in the supine position; she failed to get any score from the items involving stretching the right and left arms forwards on forearms in a prone position. She got 5 points from the sitting section in which there was no function she was able to do independently (Table 1). She got 3 points in total from the crawling and kneeling sections, and just as in the sitting section, there was no activity in these sections that she could do independently (Table 1). On the other hand, our case got 1 point from the last two items of the scale which involved standing up; yet, she could not get any point from the walking, running, and jumping section (Table 1).

The functional independence level of the case was determined by using the Pediatric Functional Independence Measure (WeeFIM). The WeeFIM evaluation gauge was formed by taking the Functional Independence Measure (FIM) as the model, which was used as the evaluation method for adult rehabilitation. This gauge is used to determine the functional independence levels of the children with cerebral paralysis and other developmental disorders as well as the changes occurring in time-dependent functions [38]. The Turkish validity and reliability study on WeeFIM was conducted by Erkin and Aybay in 2001 [39]. WeeFIM is a measurement method consisting of 18 items and 6 sections referred to as self-care, sphincter control, mobility-transfer, locomotion, communication, and cognitive function. In these sections, while the function in each item is being performed, the scoring is done from 1 to 7 according to whether or not any aid is received, the number of aids, whether or not the activity is done on time, or whether an assistive device is required or not. When the task assigned is performed by totally receiving help, then it is evaluated as 1 point, whereas when it is performed totally independently, in proper time and in a secure way, then it is evaluated as 7 points [39].

Our case got 9 points from the self-care field, which is one of the substeps of WeeFIM, whereas, she got 2 points from the sphincter control field, 4 points from the transfers field, 3 points from the movement field, 13 points from the communication field, and 15 points from the social status field (Table 2).

**Figure 1:**
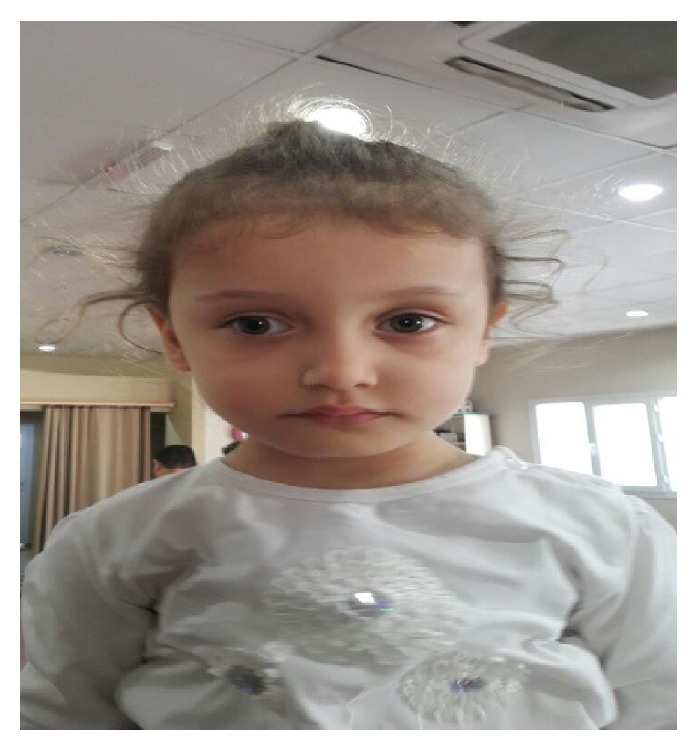
She was 32 months old.

The case was included in the physiotherapy and rehabilitation program by the physiotherapist for one hour for 5 days a week throughout the period of 13 months. In order to support the normal motor development during the treatment program, Bobath's neurodevelopmental treatment approach was applied. While the neurodevelopmental steps were being followed, the near-term and far-term targets were determined by starting from what the case was able to do. In physiotherapy practices, particular attention was paid to normalizing sensorial and motor experiences as well as forming the good posture, facilitating the balance and righting reactions, optimizing the muscular tonus, and maintaining motor control. Within the scope of neurodevelopmental treatment, again, particular attention was paid to keeping the distal and proximal segments in harmony. It was targeted that stability in the proximal segment and a proper movement in the distal segment be exposed. Within the scope of this treatment, the use of audio-visual stimulants was taken care of in the course of the activities, since motor learning was of great importance. By taking into consideration the motivation of the case, particular attention was paid to the repeating numbers of activities, their variability, and teaching the activities within the functioning process. The activities that were focused on involved turning from the supine position back to the sitting position, crawling, transferring weight, unassisted sitting, climbing up, taking steps, walking, and climbing up and down the stairs. When the case was 32 months old (Figure 1), the second examination was made by another physiotherapist. As a result of the examination, it was determined that our case was able to turn around independently from the supine position to prone position and vice versa, that she was able to lift her herself up on her forearms in the prone position, that she was able to sit on her own, and that she was also able to crawl and walk independently. During the second examination of GMFM test, our case got a full score from lying and rolling and sitting sections, all the activities of which she was able to perform independently. In the meantime, she got 38 points from the crawling and kneeling sections, 30 points from the standing section, and 31 points from the walking, running, and jumping sections (Table 3). On the other hand, as for the substeps of WeeFIM, she got 12 points from the self-care field, 2 points from the sphincter control field, 11 points from the transfers and movement fields, 13 points from the communication field, which was the same as the initial value, and 16 points from the item, social status (Table 3). The written informed consent form was received from her mother for the publication of this case report and any accompanying images.

## 3. Discussion

JS is a rare autosomal recessive disorder characterized by cerebellar vermis hypoplasia [40]. In fetuses with JS, ultrasound examination from the 20th or 21st week of gestation can detect hypoplasia of the cerebellar vermis, which can be associated, in a subset of fetuses, with polydactyly, occipital encephalocele, or both. If a cerebellar malformation is suspected, foetal MRI will confirm the diagnosis, often allowing recognition of the molar tooth sign [14]. The basic symptoms that emerge in the first months of life are in the form of hypotonia, developmental delay, abnormal eye movements, and an altered respiratory pattern [40]. In JS, the diagnosis is vital since it is recessive and it has a recurrence risk of 25% [3]. Radiological evidence of marked cerebellar vermis abnormalities is of great importance in establishing the diagnosis of this disease [41]. In literature, studies have shown that children with JS were referred to medical center due to especially developmental delay and respiratory disorders [10, 40, 42]. In a study to identify the underlying genetic defect in three brothers with JS, a male patient 18 years old was referred to medical genetics due to mental retardation and visual impairment [43]. Gagliardi et al. have discussed a case with JS who attended cognitive rehabilitation [44]. As stated by Gagliardi et al. she has received a diagnosis of oculomotor apraxia with compensatory head movement and winks when 2 months old [44]. Our case when 4 months old has received a diagnosis with her mother realizing that the baby could not follow the objects. When the literature is reviewed, the studies over JS are seen to be rather towards clinical symptoms and diagnosis. Arora discussed the clinical characteristics of a 12-year-old male case in 2014. The case was determined to have had growth and mental retardation and weakness in two of the lower extremities along with ataxia symptoms as well as an abnormal facial expression [45]. Akhondian et al. reported three siblings with similar clinical features, including developmental delay, mental retardation, and ophthalmologic disorders [40]. Various studies which discussed case with JS have described especially in the first years of life hypotonia, developmental delay, deficits in speech and language development, and visual problems and are in literature [42, 43, 46]. Another sign which indicates attention in cases with JS is early smiling [10, 47]. Choh et al. reported that cases' MRI brain which showed typical features of JS [48]. In our case, the diagnosis is definite sight of molar tooth. Some results include hypotonia, developmental retardation, nystagmus, and poor language skills learned from her mother. When our case was 12 months old, she was made to start training in a private educational institution due to developmental delay, and afterwards, she was made to continue her physiotherapy and rehabilitation program in a private medical center when she was 19 months old. Prior to the commencement of the treatment, our case, who had only movements such as head control, assisted sitting, and turning right and left, was followed up through the physiotherapy and rehabilitation program for 13 months. Akhodian et al. reported that siblings could hold their heads steady while sitting at average age of one year and could sit independently at age of two years. They walked alone in the age of four years. Similarly bowel and bladder control emerged at age of four years [40]. Chafai-Elalaouni et al. reported that their case with JS walked in age of 2 between 4 years [43]. Gagliardi et al. presented a girl with JS who has developmental delay together with poor muscular tone. Independent walking was reached at 2 years and 6 months, however accompanied by mild gait ataxia. Clumsiness in both fine and gross motor abilities was evident. Language development was mildly delayed. Her first words were in place by the expected age, two words sentences at 36 months [44]. In literature, this study is the most comprehensive study in terms of rehabilitation outcomes. The case attended rehabilitation programme was tailored and adapted all along the intervention since she was 6 months old. As stated by Gagliardi et al. visual perceptual and sequential skills were scarcely affected by the extensive rehabilitation program [44]. In Arora's study in which it was emphasized that the treatment of JS would be optimized through a multidisciplinary approach, it was stated that proper medication and cognitive and behavioural rehabilitation were important for the respiratory problems of the newborn baby in particular [45].

**Figure 2:**
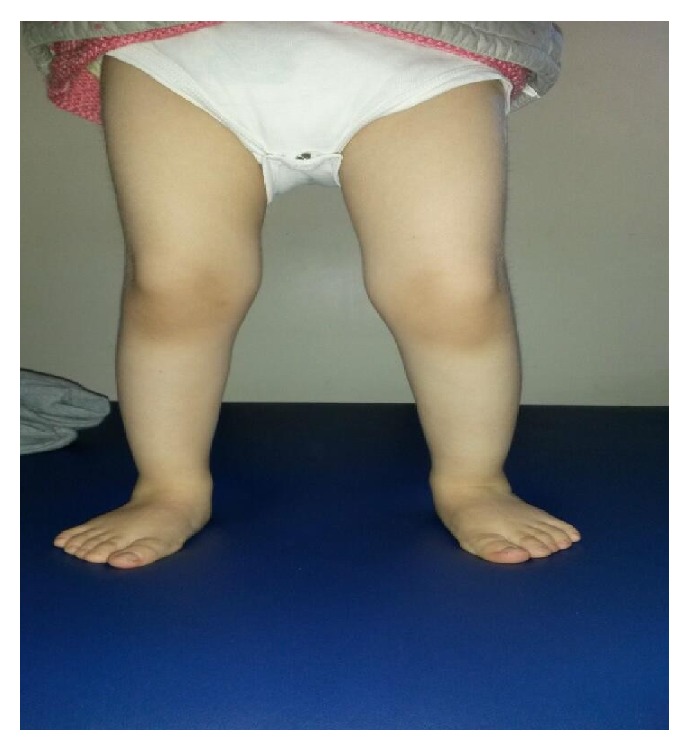
Her standing position (wide gait).

In our study, we wanted to show the positive effects of physiotherapy practices on motor development, along with the JS case we have presented. In the wake of the studies we conducted along with our case, we observed the fact that functionality could be enhanced through physiotherapy practices, that motor development could be supported, and that the child involved could become more independent and active. Our 32-month-old case got 51 points from lying and rolling section of GMFM in terms of motor development at the end of 13-month treatment. In this section, she got a full score from the items, which were turning from the supine position to the prone position by rolling towards right and left, stretching the right and left arms forwards from the prone position on forearms, and also lifting the chest up with an elbow extension while in the same position. In the sitting section, starting from sitting along with head control up until the last item, which was the activity of sitting in a higher seat on the floor, she got a full score. As for the section crawling and kneeling, she, again, got full score, except for the items involving climbing down 4 steps by crawling backwards on hands and knees and walking 10 steps on knees without the support of her arms. She also got full score from the standing section of the test, which involved the activities holding on to a higher seat and standing up, staying still for 20 sec without the support of arms, and standing up without using the arms while sitting in a lower seat. As for the functions of controlled sitting on the floor without arm support, kneeling down, and taking an object from the floor, she almost reached the level of partially completing these activities. In the functions of walking, running, and jumping, which were included in the final section of the test, she was able to walk 10 steps forwards with an object in her hand and perform the activities like kicking the ball with the right and left feet independently. While she partially completed the activities that involved taking 5 steps in the right and left directions while her arms were up and climbing up 4 stairs by changing the steps while holding on to a single handrail, she was independently able to start the activities that involved walking in 2 cm width and climbing down 4 stairs by changing the steps while holding on to a single handrail. Our case did not get any score from this section of the test that involved the activities of jumping over a stick at a knee level with the right and left feet, jumping 30 cm upwards with both feet, and climbing up and down the stairs without holding on to the handrail. As for the functional independence level of our case; according to WeeFIM results, she was able to reach the level of having a meal with minimal help in the self-care section, performing a modified independent movement towards the chair in the transfer section, and climbing up the stairs and walking independently under supervision in the movement section. At the end of 13-month period of treatment there was no change in our case's language development. She can still say a word. She still has urine and stool incontinence. She has not have sphincter control. She has wide gait (Figure 2). According to our observations, she is a child who is well adjusted and calm in social terms. There was any record of the first 6-month period when physiotherapy and rehabilitation programmes were started. So, this is the greatest limitation in our study. The second limitation is that it is not possible to compare our findings because there is not any study including detailed physiotherapy and rehabilitation program for support motor development in cases with JS and follow-up results discussed in the literature.

Therefore, the magnitude of the progress achieved following our physiotherapy and rehabilitation practices is not clear. The third limitation is that no formal speech assessment or IQ assessment was performed during the study.

Today, it is still not possible to treat JS but it is possible to offer prenatal diagnosis and genetic counseling to those who have been confirmed by gene mutation. And the results of this study also have shown that our case with JS benefits from programme of neurodevelopmental physiotherapy and rehabilitation that support motor development. So we are of the opinion that physiotherapy and rehabilitation programs will improve normal motor development and functionality during the treatment processes of JS and physiotherapists would contribute to the patient's functional recovery by applying a patient-tailored treatment programme.

## Figures and Tables

**Table 1:** First evaluation scores of Gross Motor Function Measure (GMFM).

Dimensions	Score
Lying and rolling	20
Sitting	5
Crawling and kneeling	3
Standing	1
Walking, running and jumping	0

**Table 2:** First evaluation scores of Functional Independence Measure (WeeFIM).

Dimensions	Score
Self-care	9
Sphincter control	2
Mobility-transfer	4
Locomotion	3
Communication	13
Cognitive function	15

**Table 3:** Outcome of tests scores (GMFM and WeeFIM).

GMFM			WeeFIM		
Dimension	First	End	Dimension	First	End
Lying and rolling	20	51	Self-care	9	12
Sitting	5	60	Sphincter control	2	2
Crawling and kneeling	3	38	Mobility-transfer	4	11
Standing	1	30	Locomotion	3	11
Walking, running and jumping	0	31	Communication	13	13
			Cognitive function	15	16
*Total*	*29*	*210*	*Total*	*46*	*65*

## References

[1] Maria B. L., Boltshauser E., Palmer S. C., Tran T. X. (2016). Clinical features and revised diagnostic criteria in joubert syndrome. *Journal of Child Neurology*.

[2] Joubert M., Eisenring J. J., Robb J. P., Andermann F. (1969). Familial agenesis of the cerebellar vermis: a syndrome of episodic hyperpnea, abnormal eye movements, ataxia, and retardation. *Neurology*.

[3] Doherty D., Millen K. J., Barkovich A. J. (2013). Midbrain and hindbrain malformations: advances in clinical diagnosis, imaging, and genetics. *The Lancet Neurology*.

[4] Gleeson J. G., Keeler L. C., Parisi M. A. (2004). Molar tooth sign of the midbrain-hindbrain junction: occurrence in multiple distinct syndromes. *American Journal of Medical Genetics*.

[5] Parisi M. A., Doherty D., Chance P. F., Glass I. A. (2007). Joubert syndrome (and related disorders) (OMIM 213300). *European Journal of Human Genetics*.

[6] Boltshauser E., Isler W. (1977). Joubert syndrome: episodic hyperpnea, abnormal eye movements, retardation and ataxia, associated with dysplasia of the cerebellar vermis. *Neuropadiatrie*.

[7] Saraiva J. M., Baraitser M. (1992). Joubert syndrome: a review. *American Journal of Medical Genetics*.

[8] Valente E. M., Brancati F., Dallapiccola B. (2008). Genotypes and phenotypes of Joubert syndrome and related disorders. *European Journal of Medical Genetics*.

[9] Maria B. L., Quisling R. G., Rosainz L. C. (1999). Molar tooth sign in Joubert syndrome: clinical, radiologic, and pathologic significance. *Journal of Child Neurology*.

[10] Andermann F., Andermann E., Ptito A., Fontaine S., Joubert M. (1999). History of Joubert syndrome and a 30-year follow-up of the original proband. *Journal of Child Neurology*.

[11] Sönmez F., Güzünler-Şen M., Yılmaz D. (2014). Development of end-stage renal disease at a young age in two cases with joubert syndrome. *Turkish Journal of Pediatrics*.

[12] Badano J. L., Mitsuma N., Beales P. L., Katsanis N. (2006). The ciliopathies: an emerging class of human genetic disorders. *Annual Review of Genomics & Human Genetics*.

[13] Hildebrandt F., Benzing T., Katsanis N. (2011). Ciliopathies. *The New England Journal of Medicine*.

[14] Romani M., Micalizzi A., Valente E. M. (2013). Joubert syndrome: Congenital cerebellar ataxia with the molar tooth. *The Lancet Neurology*.

[15] Lancaster M. A., Gopal D. J., Kim J. (2011). Defective Wnt-dependent cerebellar midline fusion in a mouse model of Joubert syndrome. *Nature Medicine*.

[16] Chizhikov V. V., Davenport J., Zhang Q. (2007). Cilia proteins control cerebellar morphogenesis by promoting expansion of the granule progenitor pool. *Journal of Neuroscience*.

[17] Spassky N., Han Y.-G., Aguilar A. (2008). Primary cilia are required for cerebellar development and Shh-dependent expansion of progenitor pool. *Developmental Biology*.

[18] Garcia-Gonzalo F. R., Corbit K. C., Sirerol-Piquer M. S. (2011). A transition zone complex regulates mammalian ciliogenesis and ciliary membrane composition. *Nature Genetics*.

[19] Brailov I., Bancila M., Brisorgueil M.-J., Miquel M.-C., Hamon M., Vergé D. (2000). Localization of 5-HT6 receptors at the plasma membrane of neuronal cilia in the rat brain. *Brain Research*.

[20] Händel M., Schulz S., Stanarius A. (1999). Selective targeting of somatostatin receptor 3 to neuronal cilia. *Neuroscience*.

[21] Ferland R. J., Eyaid W., Collura R. V. (2004). Abnormal cerebellar development and axonal decussation due to mutations in AHI1 in Joubert syndrome. *Nature Genetics*.

[22] Parisi M. A. (2009). Clinical and molecular features of Joubert syndrome and related disorders. *American Journal of Medical Genetics, Part C: Seminars in Medical Genetics*.

[23] Merritt L. (2003). Recognition of the clinical signs and symptoms of joubert syndrome. *Advances in Neonatal Care*.

[24] Juric-Sekhar G., Adkins J., Doherty D., Hevner R. F. (2012). Joubert syndrome: Brain and spinal cord malformations in genotyped cases and implications for neurodevelopmental functions of primary cilia. *Acta Neuropathologica*.

[25] Yachnis A. T., Rorke L. B. (1999). Neuropathology of Joubert syndrome. *Journal of Child Neurology*.

[26] Friede R. L., Boltshauser E. (1978). Uncommon syndromes of cerebellar vermis aplasia. I: joubert syndrome. *Developmental Medicine and Child Neurology*.

[27] Poretti A., Boltshauser E., Loenneker T. (2007). Diffusion tensor imaging in Joubert syndrome. *American Journal of Neuroradiology*.

[28] Spampinato M. V., Kraas J., Maria B. L., Walton Z. J., Rumboldt Z. (2008). Absence of decussation of the superior cerebellar peduncles in patients with Joubert syndrome. *American Journal of Medical Genetics, Part A*.

[29] Engle E. C. (2010). Human genetic disorders of axon guidance.. *Cold Spring Harbor perspectives in biology*.

[30] Elhassanien A. F., Alghaiaty H. A.-A. (2013). Joubert syndrome: Clinical and radiological characteristics of nine patients. *Annals of Indian Academy of Neurology*.

[31] Paksu M. S., Dağdemir A., Taşdemir H. A., Güngör O., Küçüködük Ş., İncesu L. (2004). Joubert sendromu; olgu Sunumu. *OMÜ Tıp Dergisi*.

[32] Brancati F., Dallapiccola B., Valente E. M. (2010). Joubert syndrome and related disorders. *Orphanet Journal of Rare Diseases*.

[33] Incecik F., Hergüner M. Ö., Altunbaşak Ş., Gleeson J. G. (2012). Joubert syndrome: report of 11 cases. *Turkish Journal of Pediatrics*.

[34] Maria B. L., Boltshauser E., Palmer S. C., Tran T. X. (1999). Clinical features and revised diagnostic criteria in Joubert syndrome. *Journal of Child Neurology*.

[35] Barkovich A. J. (2000). *Pediatric Neuroimaging*.

[36] Wood E. (2000). The gross motor function classification system for cerebral palsy: a study of reliability and stabilityover time. *Developmental Medicine & Child Neurology*.

[37] Russell D. J., Rosenbaum P. L., Cadman D. T., Gowland C., Hardy S., Jarvis S. (1989). The gross motor function measure: a means to evaluate the effects of physical therapy. *Developmental Medicine & Child Neurology*.

[38] Ottenbacher K. J., Msall M. E., Lyon N. (2000). The WeeFIM instrument: Its utility in detecting change in children with developmental disabilities. *Archives of Physical Medicine and Rehabilitation*.

[39] Erkin G., Aybay C. (2001). Pediatrik rehabilitasyonda kullanilan fonksiyonel degerlendirme metodlar. *Türkiye Fiziksel Tıp ve Rehabilitasyon Dergisi*.

[40] Akhondian J., Ashrafzadeh F., Beiraghi Toosi M., Moazen N., Mohammadpoor T., Karami R. (2013). Joubert syndrome in three children in a family: a case series. *Iranian Journal of Child Neurology*.

[41] Klein J. L., Lemmon M. E., Northington F. J. (2016). Clinical and neuroimaging features as diagnostic guides in neonatal neurology diseases with cerebellar involvement. *Cerebellum & Ataxias*.

[42] Torres M. C., Buceta M. J., Cajide M. C. (2001). Development of a child with joubert syndrome. *The Spanish journal of psychology*.

[43] Chafai-Elalaoui S., Chalon M., Elkhartoufi N. (2015). A homozygous AHI1 gene mutation (p.Thr304AsnfsX6) in a consanguineous Moroccan family with Joubert syndrome: A case report. *Journal of Medical Case Reports*.

[44] Gagliardi C., Brenna V., Romaniello R. (2015). Cognitive rehabilitation in a child with joubert syndrome: developmental trends and adaptive changes in a single case report. *Research in Developmental Disabilities*.

[45] Arora R. (2014). Joubert syndrome: imaging features and illustration of a case. *Polish Journal of Radiology*.

[46] Poretti A., Dietrich Alber F., Brancati F., Dallapiccola B., Valente E. M., Boltshauser E. (2009). Normal cognitive functions in Joubert syndrome. *Neuropediatrics*.

[47] Hodgkins P. R. (2004). Joubert Syndrome: long-term follow-up. *Developmental Medicine & Child Neurology*.

[48] Choh S. A., Choh N. A., Bhat S. A., Jehangir M. (2009). MRI findings in Joubert syndrome. *Indian Journal of Pediatrics*.

